# Ileal Transposition (IT) Surgery Changing the Ultrastructure of the Transposed Segment as well as Jejunum. Histomorphometric and Electron Microscopy Analysis

**DOI:** 10.1007/s11695-017-2992-z

**Published:** 2017-11-03

**Authors:** Tomasz Sawczyn, Julia Zimmermann, Dominika Stygar, Michał Kukla, Katarzyna Nabrdalik, Natalia Matysiak, Łukasz Mielańczyk, Konrad Wojciech Karcz

**Affiliations:** 1 Department of Physiology, School of Medicine with the Division of Dentistry in Zabrze, Medical University of Silesia, Katowice, Poland; 2 Clinic of General, Visceral, Transplantation and Vascular Surgery, Hospital of the Ludwig Maximilian University, Munich, Germany; 3 Department of Gastroenterology and Hepatology, School of Medicine in Katowice, Medical University of Silesia, Katowice, Poland; 4 Department of Internal Medicine, Diabetology and Nephrology in Zabrze, Medical University of Silesia, Katowice, Poland; 5 Department of Histology and Embryology, School of Medicine with the Division of Dentistry in Zabrze, Medical University of Silesia, Katowice, Poland

**Keywords:** Metabolic surgery, Ileal transposition, Ultrastructure, Ileum, Jejunum, Transmission electron microscopy

## Abstract

**Objective:**

Ileal transposition (IT) procedure leads to higher secretion of incretin hormones what is associated with a beneficial metabolic effect. However, IT will also have an influence on the related jejunum and ileum function. The aim of this research was to investigate the morphology of the jejunum and transposed ileum with the use of light and transmission electron microscopy (TEM) in order to determine the local alternations in the intestine resulting from the transposition.

**Methods:**

Twenty male, 8-week-old, obese Zucker rats underwent IT and six of them sham surgery. To compare both groups, the transection was made at all corresponding ileum positions among both groups of animals. The ileal anastomoses among the rats of sham procedure were subsequently formed accordingly without IT. Three months following the surgery, the tissue samples of jejunum and ileum were harvested.

**Results:**

A significant increase in villus length, a decrease in the crypt depth, and an increased thickness of mucosa-muscularis-serosa (MMS) as well as cellular hyperplasia, with increased mitochondrial density of the transposed ileum segment, were observed among the group of rats which underwent IT comparing to the ones undergoing sham surgery. In rats undergoing IT, microvillus degeneration in jejunum regions was observed.

**Conclusions:**

Ileal transposition alters the morphology and ultrastructure of the ileum as well as the jejunum. Given that the microvillus membrane represents an important aspect of the enterocyte functions, a further biochemical and molecular research is necessary in order to assess whether the observed changes are beneficial or not and to explore the phenomenon of gut adaptability after metabolic surgery.

## Introduction

The current hypothesis of the diabetes remission after bariatric surgery is based on the so-called hindgut hypothesis where fast delivery of nutrients to the terminal ileum leads to a higher secretion of incretin hormones [1–3]. Based on this hypothesis, more attention was given to ileal transposition (IT). IT is a surgical procedure involving the transposition of a distal ileum segment to the proximal jejunum in a properistaltic direction with a continuity of the gastrointestinal tract. This procedure results in a fast delivery of food to the terminal ileum followed by different metabolic alterations [4–8]. The terminal ileum contains the highest density of L- and K-cells which produce incretin hormones like glukagone-like peptide-1 (GLP-1), gastric inhibitory peptide (GIP), and peptide YY (PYY) [9, 10]. Several studies describe a higher secretion of these polipeptides due to increased glucose exposition after the ileal transposition followed by an early amelioration of glucose control and remission of type 2 diabetes [4, 5, 11, 12].

The metabolic improvements originate also in the alteration of the ultrastructure of the transposed segment. It is already known from the previous studies that transposed ileal segments compared to the sham procedures present with significant hyperplasia, hyperthrophy, and-even ‘jejunization’ of transposed ileum [9, 13–16]. Regarding jejunum, the only histological changes that have been described are related to the increase in the intestinal luminal surface area and number of enteroendocrine cells [17]. In addition to the results relating to the amelioration of the glucose control, the long-term effect needs to be assessed. So far, it is not known whether the early glycaemia improvement and favourable changes in energy homeostasis are long-lasting. Similarly, not much is known if the gut morphological adaptation, which is related to this process, impairs the usual function of the intestine. Apart from the morphological changes in the transposed ileal segment, the question about changes in other parts of the intestine (especially the jejunum) and their long-term effect on benefits resulting from IT arises. To our best knowledge, there are no studies assessing the morphological alternation of the jejunum being attached to the transposed ileum performed up to date.

The aim of this research was to investigate the morphology of the transposed ileum as well as the jejunum with the use of light and electron microscopy (TEM), to determine local alternations in the intestine resulting from the ileal transposition.

## Methods

### Animals

All experimental procedures received the approval of the Local Ethical Committee for Experiments on Animals. Twenty male, 8-week-old, obese Zucker (Crl:ZUC-Lepr ^fa^) rats underwent a surgical procedure. They were purchased from Charles River Breeding Laboratories (Wilmington, Mass), and their initial weight oscillated between 250 and 275 g. The animals were housed in plastic cages under controlled conditions (temp. 23 ± 0.3 °C; 12 h light, 12 h dark cycle, lights on 6:00 AM to 6:00 PM, humidity 70 ± 1%).

### Ileal Transposition and Sham Surgery

Following the standard abdominal midline incision of 4–5 cm, Bauhin’s valve was identified. The 50% of distal ileum was localized and transected. The first anastomosis was subsequently formed as an end-to-end ileoileostomy in order to restore ileal continuity, excluding the transposed segment. All anastomoses were performed as interrupted end-to-end extramucosal anastomoses using PDS 6/0 (Ethicon, Blue Ash, Oh). Subsequently, the ligament of Treitz was identified, and the jejunum was divided 5 cm aborally. After this, the ileal segment was inserted in an isoperistaltic fashion, forming two end-to-end anastomoses.

For sham surgery, transections were made at all three corresponding positions. Anastomoses were subsequently formed accordingly however without IT.

Fascia and skin closure were conducted as a continuous suture using Monocryl 4/0 and Vicryl 4/0. After the operation, animals were maintained on liquid diet for 24 h (Nutrison, Nutricia, Poland).

### Tissue Collection

Three months after surgery, in order to collect the tissue, an anaesthesia was induced and maintained using isoflurane 2% and oxygen flow at 2 L/min under spontaneous breathing. Animals were sacrificed and tissues were harvested. The length of collected segments of the intestine did not differ between the two groups. The closest part of the jejunum directly after the interposed segment of ileum in the IT group and the closest parts of the jejunum incision in the sham group were chosen for histological analysis. All segments of the transposed ileum, the corresponding ileum of the sham group, and the jejunum segments in both groups were analysed and compared.

### Histomorphometry Analysis of the Ileum and Jejunum Segments

Histomorphometric analysis was performed using the light microscope with an optical reticule and micrometre at 10× magnification. The whole transposed segment of the ileum as well as jejunum and anatomically corresponding segments of the sham group were explanted and rinsed with cold phosphate-buffered saline (PBS). The tissues were fixed in formalin, embedded in paraffin, and sectioned along the villus axis. To assess the maximum villi length (measured from above the crypt to the tip of the villus), crypt depth, and submucosa/muscularis/serosa thickness, two sections of intestine were taken from each animal. Intestine sample, haematoxylin, and eosin staining was performed after. Seven to eight slides were analysed.

### TEM Analysis of the Jejunum and Ileum Segments

TEM analyses were made using TEM Imaging and Analysis software (FEI). One section of the intestine was taken from each animal. The quantitative, structural information (microvillus length, mitochondria number/one enterocyte) was obtained from ten samples of each section.

Immediately after collection, the ileal and jejunal segments were fixed in 2.5% glutaraldehyde (SERVA Electrophoresis GmbH—Heidelberg, Germany) in cacodylate buffer (pH 7.4) for 2 h at room temperature and then washed several times in the same buffer. Subsequently, the tissue was post-fixed in 1% osmium tetroxide (Polysciences Inc., Niles, Illinois, USA) and dehydrated in a graded series of ethanol (50, 70, 90, and 96%) and propylene oxide. The samples were then infiltrated in 2:1 (*v*:*v*) and 1:2 (*v*:*v*) propylene oxide/Epon 812 mixtures, embedded in Epon 812 epoxy resin (SERVA Electrophoresis GmbH—Heidelberg, Germany), then polymerized for 48 h at 60 °C. Ultrathin sections were cut from representative samples with a diamond knife (450; RMC, Tucson, USA) using a Reichert OmU-3 ultramicrotome (Reichert, Vienna, Austria), mounted on 300-mesh copper grids, and stained with 0.5% aqueous uranyl acetate and lead citrate (LAURYLAB Saint—Fons, France) using a Leica EM AC 20 stainer (Leica Microsystems, Vienna, Austria). After air drying of the grids, they were examined in a TECNAI™ G2 12 Spirit BioTWIN transmission electron microscope (FEI, Eindhoven, The Netherlands) at 120 kV. Images from representative regions were captured with a Morada CCD camera (Olympus Soft Imaging System Solutions GMBH, Münster, Germany).

### Statistical Analysis

Values are presented as mean ± standard deviations (SD). Tukey’s test, ANOVA, analyses were performed using Statistical 10.0. Dixon’s *Q*-test was performed to detect significant outliers. The level of statistical significance was set as *p* < 0.05.

## Results

The comparison of intestine ultrastructure between the IT and the sham group assessed after 3 months following the surgery indicated significant differences in the morphology of the ileum and the jejunum.

### Ileum Histomorphometry Analysis

**Fig. 1:**
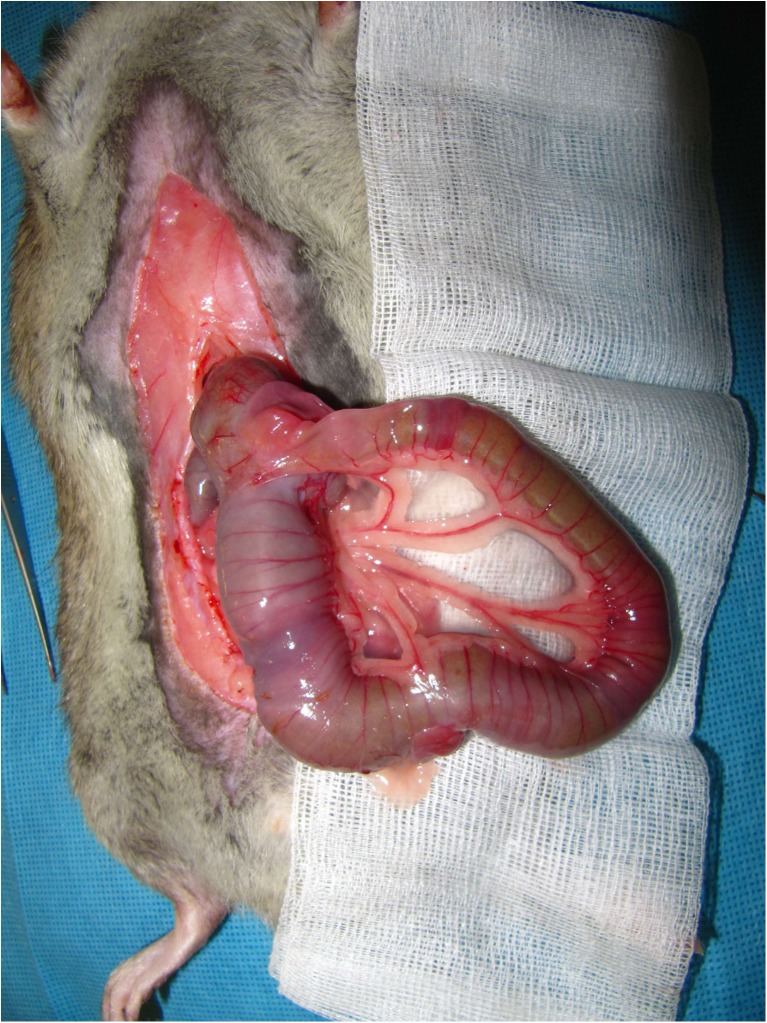
Hypertrophy of ileal transposed segment. Male obese Zucker rats 3 months after ileal transposition (IT). A standard abdominal midline incision of 4–5 cm was used to gain abdominal access

**Fig. 2:**
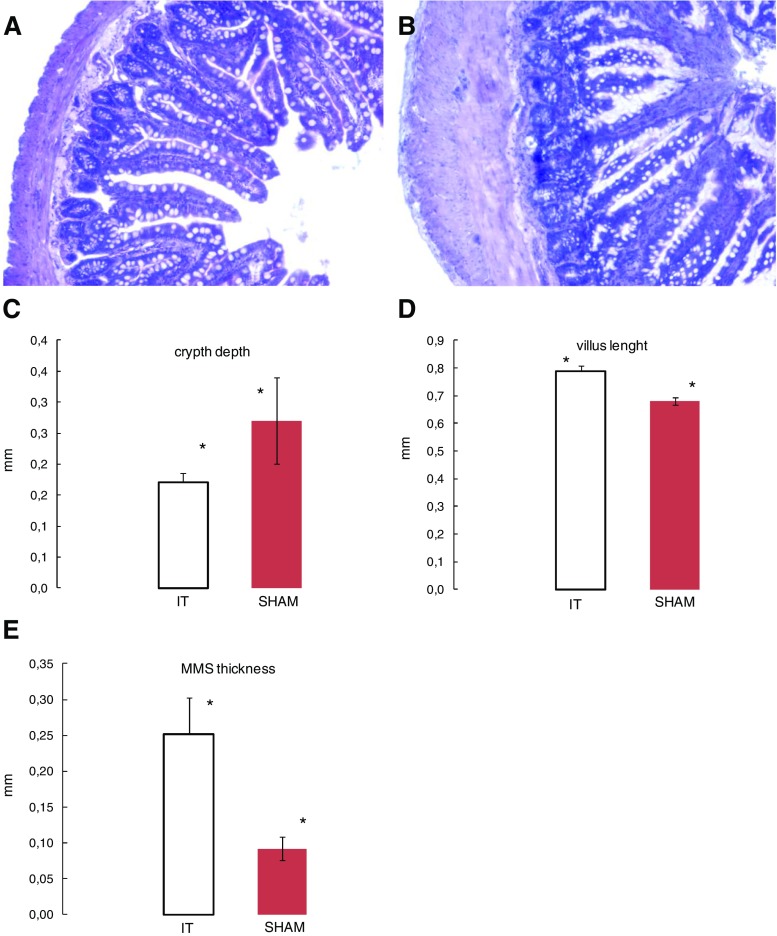
Histomorphological analysis of villi length, crypt depth and mucosa, muscularis, and serosa (MMS) thickness in SHAM (**a**) and IT (**b**). The microscopic pictures represent the structure of the ileum. From left to right: serosa, muscularis and mucosa, followed by the crypt and villus. *Significant differences, by Tukey’ test **c** *p* < 0.016, **d** *p* < 0.017, and **e** *p* < 0.0001

Regarding ileum alterations, the macroscopic hypertrophy has been observed firstly (Fig. 1). Then, a significant enlargement of villus length in the IT group compared to the rats has been revealed (0.68 ± 0.14 vs. 0.79 ± 0.02 mm, *p* < 0.016; Fig. 2a, c). Furthermore, the SHAM group showed a significant deeper crypt (0.27 ± 0.07 vs. 0.17 ± 0.015 mm, *p* < 0.017; Fig. 2b, d). In contrast, the observed MMS (mucosa, muscularis, and serosa) area of the IT segment appeared to be significantly thicker than the comparable part of the sham operated rats (0.251 ± 0.05 vs. 0.091 ± 0.016 mm, *p* < 0.0001; Fig. 2a, b, and e).

**Fig. 3:**
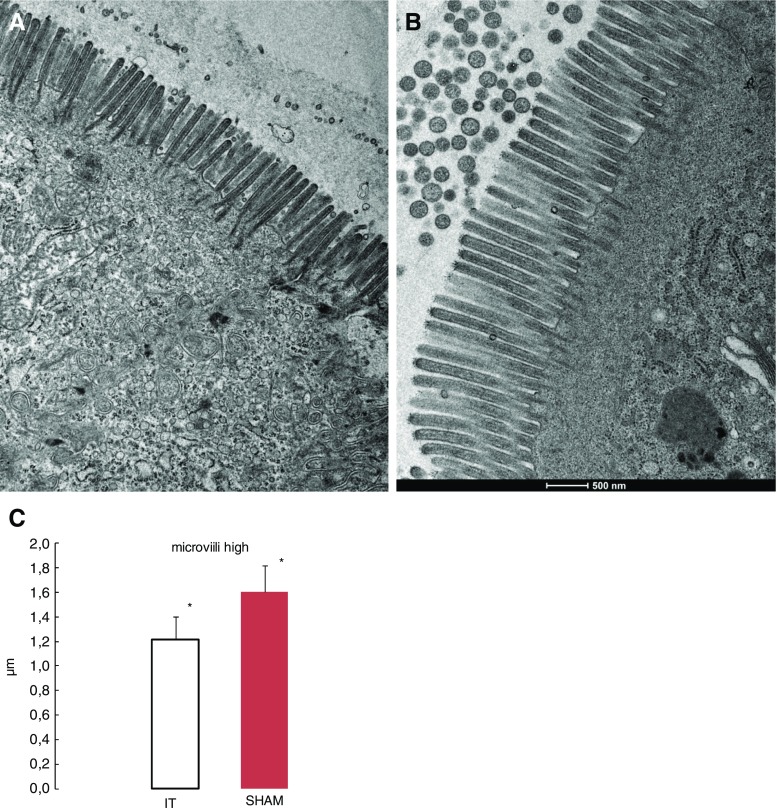
Electron micrograph of the segments of ileum mucosa. IT group transposed segment (**a**). SHAM group—ileum mucosa (**b**). *Significant differences by Tukey’s test *p* < 0.0001 (**c**)

Ileum TEM analysis revealed that after ileal transposition, microvilli in the IT group compared to the sham group were significantly shorter (1.217 ± 0.18 vs. 1.600 ± 0.210 μm, *p* < 0.0001; Fig. 3c). Those microvilli appeared also to be irregular (Fig. 3a), whereas the sham group presented with regular microvilli combined with a bundle of thin, striated filaments connected to the terminal web (Fig. 3b).

### Ileum Enterocyte Mitochondrial Density Analysis

**Fig. 4:**
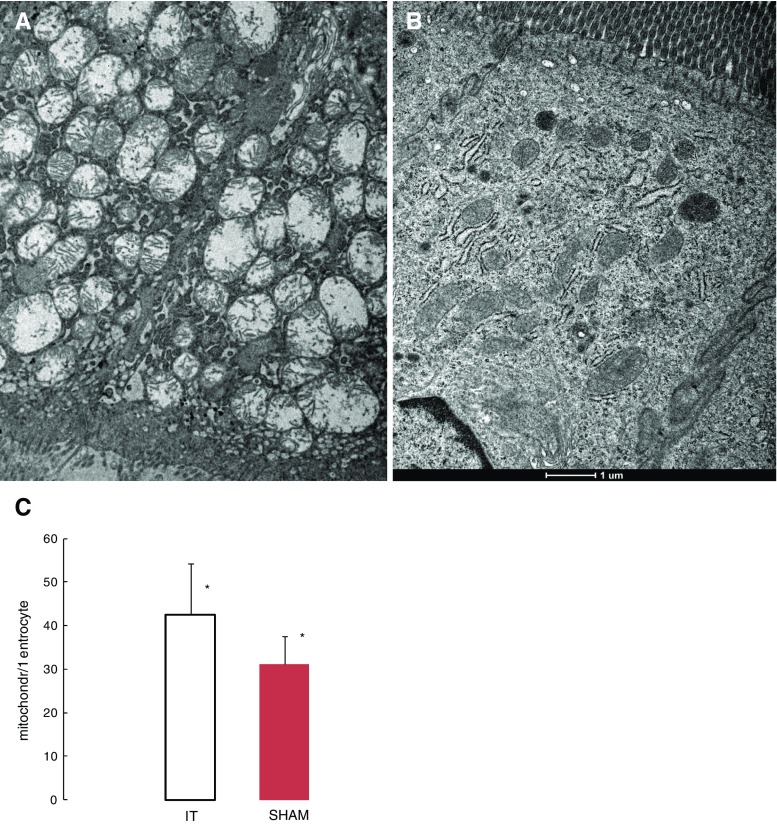
Electron micrograph of apical part of the ileum enterocyte cytoplasm. The apical cytoplasm of the IT group (**a**). The apical cytoplasm of the SHAM group (**b**). The ileum mitochondria number per enterocyte in IT- and SHAM-operated rats 3 months after surgery (**c**). *Significant differences by Tukey’s test *p* < 0.0014

The apical part of the transposed ileum enterocyte cytoplasm contains markedly swollen mitochondria with short cristae. Most of these mitochondria have prominent spaces in the matrix and the endoplasmic reticulum (ER) which appear to be dilated (Fig. 4a). With regard to the sham group (Fig. 4b), a smaller number of mitochondria and various sharps were observed (31.06 ± 6.58 vs. 45.55 ± 11.71 mitochondia/enterocyte, *p* < 0.001; Fig. 4c).

### Jejunum Histomorphometry Analysis

**Fig. 5:**
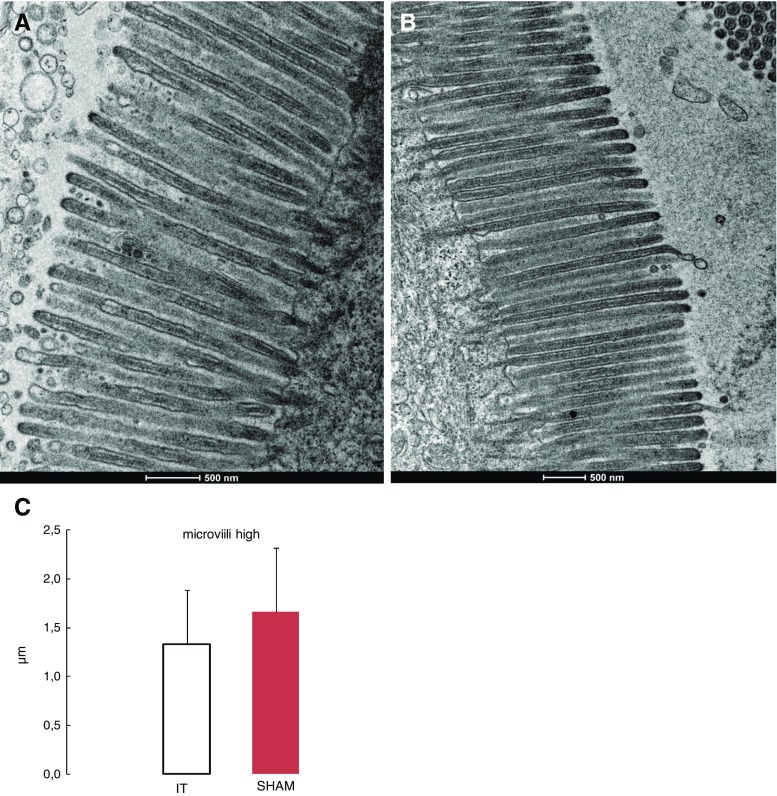
Electron micrograph of apical part of the jejunum. IT group (**a**). SHAM group (**b**). The jejunum microvilli high in operated rats 3 months after surgery are not significant. Tukey’s test *p* ≤ 0.05 (**c**)

The TEM jejunum microvillus height comparison of the IT and sham group presents similar results (1.326 ± 0.55 vs. 1.66.55 ± 0.654 μm, *p* = 0.101; Fig. 5c). The observation of the IT group indicates an altered jejunum brush border and an irregular microvillus membrane. The microvillus tips are more or less swollen and a vesication of the microvilli was observed (Fig. 5a). The sham group was normally organized in microvilli (Fig. 5b).

**Fig. 6:**
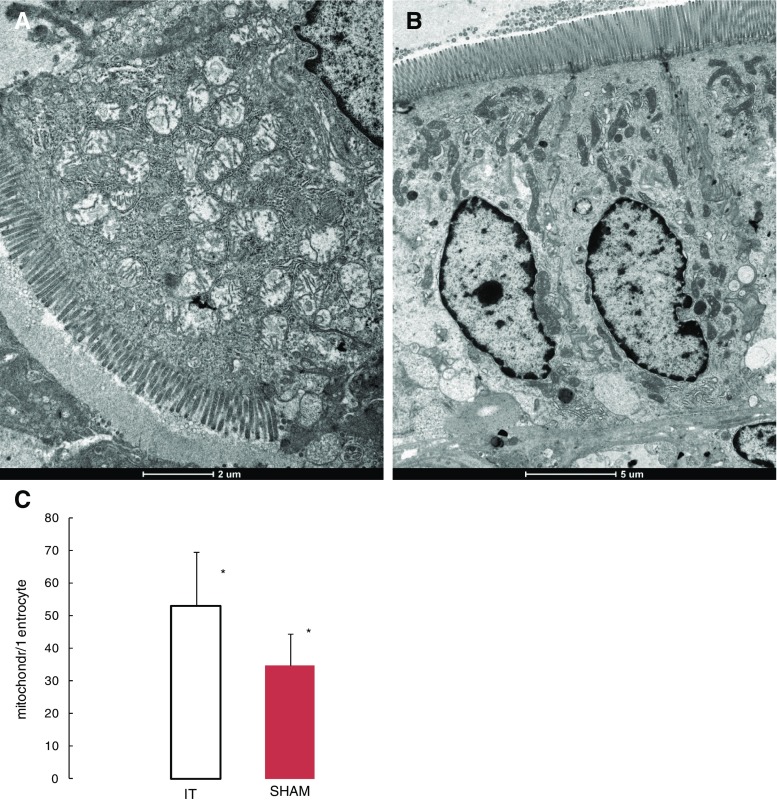
Electron micrograph of the apical part of the transposed jejunum enterocyte cytoplasm. IT group cytoplasm (**a**). SHAM group cytoplasm (**b**). The jejunum mitochondria number per enterocyte in IT- and SHAM-operated rats 3 months after surgery (**c**). *Significant differences by Tukey’s test, *p* = 0.0047

The comparison of the mitochondrial number per enterocyte in the jejunum shows similar changes as observed in the ileum segment. The number of mitochondria per enterocyte is significantly higher in the IT group compared to the sham group (53.00 ± 16.42 vs. 34.76 ± 9.70 mitochondia/enterocyte, *p* < 0.0047; Fig. 6c). The apical part of the enterocyte cytoplasm of the IT group contains markedly swollen mitochondria with short cristae (Fig. 6a). In comparison, the sham group features varied mitochondria shapes, and the luminal surface is completely covered with regular microvilli (Fig. 6b).

## Discussion

Our study is the first to observe the morphological changes in the jejunum as a result of the ileum transposition. The TEM analysis of the jejunum shows no differences in the microvillus height between the IT and SHAM group. Nevertheless, the IT group shows altered jejunum brush border and an irregular microvillus membrane. The microvillus tips were swollen and a vesication of the microvilli was observed. The most important physiological role of the jejunum is absorption, which is combined with the level of microvillus density. Altered microvilli may result in the decreased functioning of the jejunum with unpredictable consequences.

Because the closest part of the jejunum directly following the interposed segment of ileum was chosen for histological analysis, it is difficult to assess general changes of the jejunum after the IT. Nevertheless, the described changes occurred frequently and the differences between two studied groups of rats were significant. Surprisingly, in this part of the jejunum, we also observed increased mitochondrial density in the IT-operated rats compared to the anatomically corresponding segment from the SHAM group. While in the case of the ileum, an increased mitochondrial density could be associated with an elevated absorption of glucose; in the case of jejunum, it is difficult to determine the probable cause.

Regarding the morphological alternation of the transposed ileum segment, we speculated in our former studies that it undergoes a hypertrophy and further ‘jejunization’ caused by the constant nutrient overstimulation, which eventually impairs its incretin-secreting ability [13]. Results of the presented study indicated a significant increase in villus length and a decrease crypt depth, as well as increased MMS thickness of interposed ileum in the IT group of rats, which is consistent with the other studies performed up to date [13, 17–19]. The TEM shows hyperplasia of ileum enterocytes combined with decreased microvillus length and a considerable increase in the number of enterocytes’ mitochondria. It is hypothesized that the increased mitochondrial density is correlated with a higher level of glucose absorption [18]. Moreover, markedly swollen mitochondria with short cristae and prominent spaces in the matrix with dilated ER may indicate a significantly increased enterocyte metabolism. The mitochondria damage could result in decreased energy production and ultimately may lead to the many cell dysfunctions. On the other hand, the intensified metabolism in the enterocytes may stimulate the formation of free radicals what may contribute to the increase in the cellular damage [21].

The adaptive responsiveness of the interposed ileal segment is still difficult to explain, but several changes seem to be involved in this process. Kohli et al. [19] interpreted the metabolic benefits as originating from an increased bile acid recycling. They conclude that the interposed ileum increased its surface area, but retained its bile acid absorptive capacity, while increasing the density of enteroendocrine cells. Jurowich et al. [18] proposed that the amount of sodium glucose transport proteins type 1 (SGLT1)-mediated glucose uptake in the interposed ileum increased twofold due to an increase of total SGLT1 protein at the luminal surface. The upregulation in the IT is reaching the same level of SGLT1 protein as in the jejunum; consequently, the improvement of the glycaemic control by metabolic surgery does not require decreased glucose absorption in the transposed ileum. However, IT results in higher ileal exposition for nutrients, including glucose, followed by ileum hypertrophy. Hypertrophy results in a L-cell hyperplasia in the interposed intestine, which is followed by an increased secretion of the antidiabetic hormones GLP-1 and PYY [20].

Nowadays, the clinical effect of observed morphological changes of intestine may be only a matter of suspicion. We suppose that morphological changes are directly linked to local metabolic activity of the transposed segment as well as with its hormonal activity, and the adaptation capability of the transposed segment may determine the limits of the durability and effectiveness of the operation performed.

Even though the IT raise a lot of interest due to its good metabolic effect on blood glucose control, there is still insufficient knowledge about the adverse effects of it on the organ itself and the whole organism. It is necessary to perform long-term studies to clarify whether the changes in the intestine structure as a consequence of IT surgery can be associated with unfavourable changes.

## Conclusion

Ileal transposition alters the morphology and ultrastructure of both the ileum and the jejunum. The current study restates the findings of ileal hypertrophy after the IT surgery and demonstrates for the first time the significant influence of this surgery procedure on the jejunum morphology.
